# The impact of over 80 years of land cover changes on bee and wasp pollinator communities in England

**DOI:** 10.1098/rspb.2015.0294

**Published:** 2015-05-07

**Authors:** Deepa Senapathi, Luísa G. Carvalheiro, Jacobus C. Biesmeijer, Cassie-Ann Dodson, Rebecca L. Evans, Megan McKerchar, R. Daniel Morton, Ellen D. Moss, Stuart P. M. Roberts, William E. Kunin, Simon G. Potts

**Affiliations:** 1 Centre for Agri-Environmental Research, School of Agriculture, Policy and Development, University of Reading, Reading RG6 6AR, UK; 2 Institute of Integrative and Comparative Biology, University of Leeds, Leeds LS2 9JT, UK; 3 Naturalis Biodiversity Centre, 2333 CR Leiden, The Netherlands; 4 Centre for Ecology and Hydrology, Lancaster LA1 4AP, UK

**Keywords:** historical land cover change, pollinators, species richness, species composition, Dudley Stamp map, LCM 2007

## Abstract

Change in land cover is thought to be one of the key drivers of pollinator declines, and yet there is a dearth of studies exploring the relationships between historical changes in land cover and shifts in pollinator communities. Here, we explore, for the first time, land cover changes in England over more than 80 years, and relate them to concurrent shifts in bee and wasp species richness and community composition. Using historical data from 14 sites across four counties, we quantify the key land cover changes within and around these sites and estimate the changes in richness and composition of pollinators. Land cover changes within sites, as well as changes within a 1 km radius outside the sites, have significant effects on richness and composition of bee and wasp species, with changes in edge habitats between major land classes also having a key influence. Our results highlight not just the land cover changes that may be detrimental to pollinator communities, but also provide an insight into how increases in habitat diversity may benefit species diversity, and could thus help inform policy and practice for future land management.

## 1. Introduction

Shifts in pollinator communities and assemblages are well documented in certain regions of the world [1–5]. Major drivers of declines in pollinators that have been identified, including climate change [6,7], spread of pathogens [8], introduction of non-native plant and pollinator species [9,10], agricultural intensification [11–13], and landscape alteration [14,15]. While some studies have explored the impact of contemporary changes in landscape and land utilization on pollinator communities [16–22], long-term historical land cover change and its impacts on pollinators have yet to be quantified—primarily owing to the lack of availability of historical land cover data (until recently) and/or methods to standardize existing biodiversity data collected by volunteers. Understanding the impact of historical land changes on pollinator communities will not only add to existing knowledge, but also help inform policy and practice relating to land management for ecosystem services and food security.

The earliest known land cover map of Britain [23] has recently been digitized, and this, combined with the availability of novel statistical methods that enable comparison of species richness data with varying sampling effort [5,24,25], finally allows study of the impacts of historical land cover on pollinator communities. While previous studies have used space-for-time substitution [26,27], our study is unique in being the first to test directly whether land cover change across multiple sites has had a significant role to play in pollinator dynamics in England. Long-term data on aculeate hymenopterans are rare, but data from the 1800s onwards have been collated and validated by the Bees, Wasps and Ants Recording Society (BWARS; www.bwars.com). Using the BWARS data, which have been standardized for taxonomic accuracy (including revisions of species names), we assess whether there have been shifts in these communities and, if so, test whether these shifts are associated with changes in land cover.

We predicted that the most substantial shifts in pollinator communities should occur in landscapes that have experienced the greatest change in land cover at spatial scales relevant to pollinators; that specific land cover types (e.g. heathland, woodland, grassland) should prove more conducive to greater species richness and diversity than others (e.g. intensive farmland); and that increase in edge habitats between land cover types would cause greater changes in species composition owing to altered community dynamics [28]. By analysing the effect of these changes on the richness and composition of pollinators, our study offers a long-term perspective on the effects of land cover change that has implications for biodiversity conservation as well as future land management.

## 2. Material and methods

### (a) Sites, land cover data and pollinator data

The earliest digital land cover data available in England come from the Dudley Stamp land utilization survey maps of the 1930s, the ground surveys of which were carried out between 1925 and 1948 [23]. These have now been digitized and are available through the Environment Agency. More up-to-date land cover information is available from the Centre for Ecology and Hydrology's UK Land Cover Map for 2007 (LCM 2007 [29]), which was derived from the semi-automated classification of satellite images. To evaluate the effect of land cover changes on changes in bee and wasp species richness, we compared two 30-year time periods (1921–1950 versus 1983–2012), corresponding to the historical and current land cover maps available, while guaranteeing that the two time periods were well separated.

**Figure 1.**
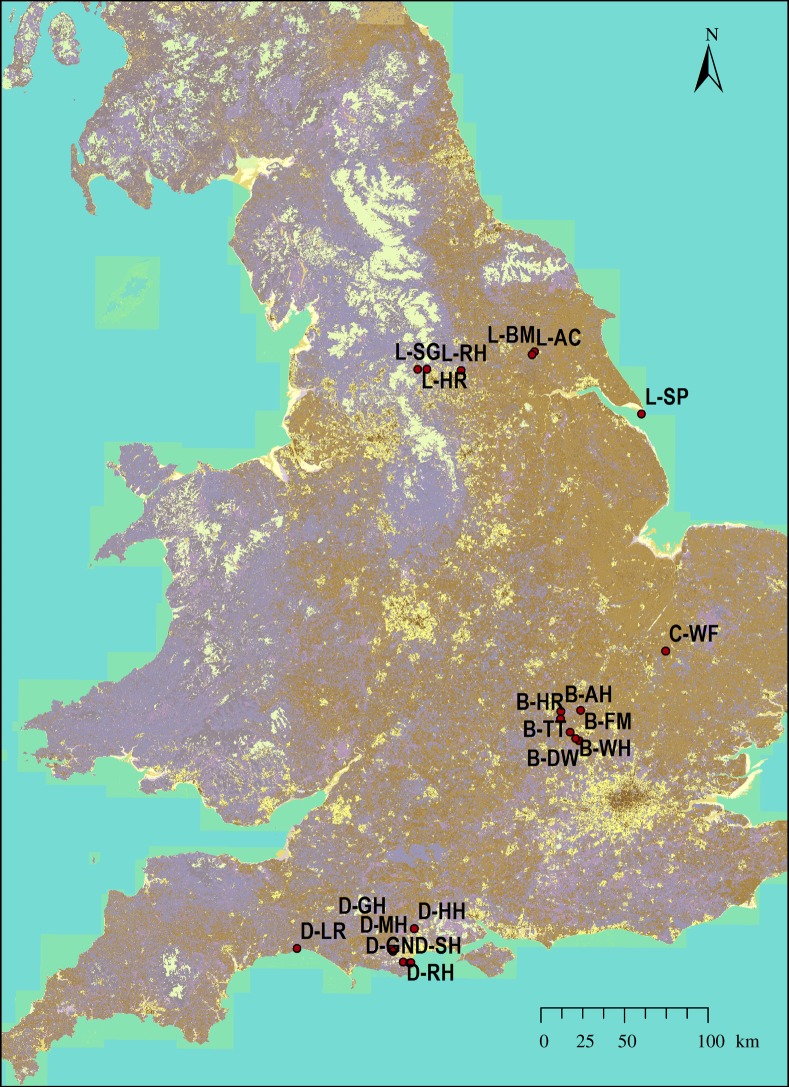
Map showing the locations of the 20 study sites within England. (Online version in colour.)

Historical data on bees and wasps were obtained from the digitized database of BWARS (www.bwars.com). From this database, sites that contained data on bee and wasp species occurrence for the historical period were identified and defined based on their historical boundaries. Overall, 20 sites were identified: six in Bedfordshire, one in Cambridgeshire, seven in Dorset and six in Yorkshire (location map of sites given in figure 1 and details of each site provided in electronic supplementary material, table S1). Existing data from 1983 onwards from the BWARS database including our fieldwork data from 2011 and 2012 (details in electronic supplementary material) constituted the current pollinator data for analysis.

### (b) Analyses of changes in land cover

The digitized Dudley Stamp map has eight major land cover categories, whereas the LCM 2007 has 23 categories, based upon UK Biodiversity Action Plan broad habitats [29]. The broad habitat categories of the LCM 2007 were re-classified to match the Dudley Stamp map categories, with an extra category being added for land cover types that did not fit into known Dudley Stamp categories (details in electronic supplementary material). Using the reclassified raster versions of both maps, the percentage change in each broad land cover category was calculated at different spatial scales: within focal sites and at 1, 2 5 and 10 km radii outside each site. These spatial scales were chosen in order to account for the typical pollinator foraging distances from a site (1 and 2 km) [30–32] and also to provide a landscape background (5 and 10 km). Spatial analyses were carried out in ArcGIS v. 10.0 [33]. Each of the percentage changes in land cover were then multiplied by a pollinator suitability score as compiled based on expert opinion [34] (details in the electronic supplementary material) for that land cover type to give a weighted land cover change value for further analyses.

In order to identify changes in edge habitat, a cell adjacency matrix (defined as the tally of the number of cells adjacent between each pairwise combination of land cover types) was calculated within each site and also outside the sites at the spatial scales mentioned earlier. The difference in the cell adjacency matrix value between the historical and current time periods for each pair of land cover classes provided the value of the change in edge habitat. This analysis was performed using Fragstats v. 4.1 [35].

### (c) Analyses of species richness change

To calculate richness change between the two periods in a manner that accounted for differences in sampling effort between the historical and current time periods, we used individual-based species accumulation curves [25]. We followed the methods described by Carvalheiro *et al*. [5], and combined interpolation and extrapolation methods, so that extrapolation would only be allowed up to threefold of the real sampling effort (see [25]), using bootstrap methods to account for possible bias owing to under- or overrepresentation of singletons and doubletons in the databases (see [5]). We also *a priori* excluded sites with very poor quality of sampling (i.e. selection criteria = sites needed to have minimum five species, minimum 10 records and less than 10-fold difference in no. of records between two time periods).

We first sorted our data into (i) all bee and wasp data and (ii) bee-only data, and then applied the process described above for every site, calculating relative richness change between the historical and the current period as *X*_2_(*n*)/*X*_1_(*n*). We then applied a log transformation (hereafter termed ‘logratio’) to normalize residuals. There were insufficient data to analyse change in wasp species richness as a separate dataset.

### (d) Analyses of species composition change

To determine whether there was a turnover of species in our study sites and to evaluate changes in patterns of pollinator assemblages, we investigated how species composition across space (assessed by comparing assemblages in each site) changed over time using the βsim index described by Lennon *et al*. [36]. The change in species composition (SCC) has an upper limit of one (no species in common within a site) and a minimum of zero (sites have identical species lists). To correct for the unequal sampling effort and observer bias, this index was modified for each time period and each site based on an individual-based probabilistic approach [24], and was calculated as
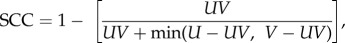
where *U* denotes relative abundance shared species in the historical period and *V* denotes relative abundance of shared species in the current period.

The sites that did not meet the selection criteria for the species richness change analysis were also excluded from the species composition change analysis. Owing to the location of the study sites within different counties, we tested for spatial autocorrelation of the land cover change data as well as the species richness change and species composition change data using the Moran's I measure in R package ‘ape’ [37] before proceeding with further analysis.

### (e) Effects of land cover changes on species richness

Weighted regression techniques were applied to test for the effects of the change in land cover on the species richness change results for each pollinator dataset. Using the rma.uni function of the R package ‘metaphor’ [38], the species richness change value for each site was weighted based on the inverse of the variance, so that cells with more reliable estimates of pollinator species richness had a higher weight in the analyses [39]. The log-ratio value obtained when calculating species richness change was used as the response variable and a null model with no explanatory variables was initially run to determine the total variability owing to heterogeneity in the data. The change in habitat suitability, the change in different edge habitats and the weighted change in each major land cover type within and around each site at varying spatial scales were then used as explanatory variables, considering also all possible two-way interactions. Changes in land cover types that were significantly correlated with each other were excluded from being in the same model (e.g. heathland and woodland). Changes in edge habitat were tested in separate models as edge density change is correlated with overall change in each habitat type. Land cover changes at different spatial scales outside of the site were tested in separate models (examples given in the electronic supplementary material). Each model was simplified using a stepwise AIC method until only the minimum adequate model remained. The models showing significant land cover change variables were then compared with the null model to determine what percentage of the existing heterogeneity could be explained by the inclusion of the explanatory variables.

### (f) Effects of land cover changes on species composition

The species composition change being an absolute value with no standard deviation meant a weighted regression could not be performed to test for the effects of land cover change. Instead, general linear models were constructed using the glm function of the R package ‘MASS’ [40], with the species composition change used as the response variable. The change in habitat suitability value, the change in edge habitat and the weighted change in each major land cover type within and around each site at varying spatial scales were then used as explanatory variables and model simplified using stepwise AIC method. Correlated changes in land cover types and changes in edge habitat were excluded from being in the same model, and land cover changes at different spatial scales outside of the site were tested in separate models. All statistical analyses were carried out in R v. 3.0.1 statistical software [41].

### (g) Accounting for possible biases

Our site selection was constrained by the availability of historical pollinator data, and therefore the sites chosen may not be representative of land cover across the country. However, we have tested land cover changes in the wider landscape (i.e. 1, 2, 5 and 10 km radii), and while our study sites are all areas of natural/semi-natural habitat (predominantly heathland), the results clearly indicate the general direction of relationships between specific land cover changes and the pollinators both within and outside these defined areas.

To check if the method following [5] completely corrected for bias owing to differences in sampling efforts, we tested the effect of the log of the relative difference in the number of records between the two time periods (Δ*R* = ln[number of records2/number of records1]). If accumulation curve estimates did not completely remove the bias owing to sampling effort (i.e. whenever Δ*R* had a significant effect on estimated richness change across sites), we calculated the partial residuals after removing the effect of sampling effort for each cell to obtain unbiased estimates of richness change for each grid cell (following Carvalheiro *et al*. [5]).

## 3. Results

### (a) Analyses of changes in land cover

The change in each major land cover category at site level and 1 km radius is given in electronic supplementary material, figure S1 and changes at 2, 5 and 10 km in electronic supplementary material, table S3. Certain categories of land cover changes were highly negatively correlated with each other. For example, heathland change and woodland change were inversely correlated at site level (*r* = −0.77, *p* < 0.001) at 1 km (*r* = −0.81, *p* < 0.001) and 2 km radii (*r* = −0.83, *p* < 0.001); grassland and arable changes were inversely correlated at site level (*r* = −0.57, *p* < 0.01), 1 (*r* = −0.59, *p* < 0.01) and 2 km radius (*r* = −0.60, *p* < 0.01); and urban and arable change were inversely correlated at the 2 km radius (*r* = −0.62, *p* < 0.01). There was considerable variation in land cover patterns among sites. Across all sites, on average, there was a within-site loss in heathland of approximately 28% (±34%) and a 13 ± 18% loss at 1 km radius. Conversely, there was an average increase of woodland within site (22 ± 27%) as well at 1 km radius (6 ± 9%). There was also an increase in arable land (average increase = 5 ± 9%) and grassland (4 ± 10%) at 1 km radius.

The edge analysis indicated that mean cell adjacency between heathland and woodland within sites increased from 47.1 ± 98.9 cells historically to 100.7 ± 128.3 cells in the current period. At 1 km radius, mean cell adjacency between heathland and woodland decreased from 471.9 ± 801.8 historically to 181.2 ± 281.9 in the current period. The mean cell adjacency between grassland and arable land within sites decreased from 56.4 ± 83.0 historically to 13.7 ± 22.7 in the current period, and from 1624.3 ± 1487.9 in the historical period to 687.7 ± 535.21 in the current period at 1 km radius.

### (b) Analyses of species richness change and species composition change

**Figure 2.**
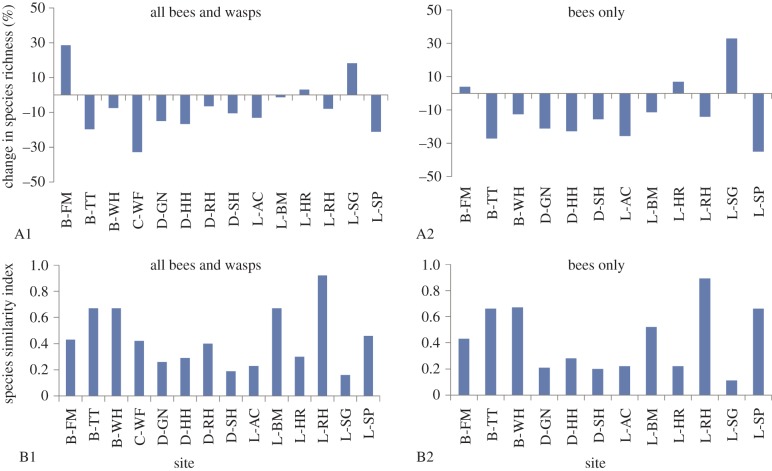
Percentage change in species richness (A1, A2) and change in species composition using Lennon index (B1, B2) at each site. (Online version in colour.)

Based on the selection criteria for quality of sampling, 14 sites met the criteria for all bee and wasp data, and 12 sites for the bee-only data. Only three sites (B-FM, L-HR and L-SG) showed an increase in species richness, whereas the rest of the sites showed declines (figure 2*a*).

The results of the species composition change analysis are given in figure 2*b*, with higher values indicating greater levels of change in composition (i.e. higher turnover of species) between the two time periods. When testing for all data (bees and wasps), four sites showed a species composition change value of over 0.5, with five sites showing values of over 0.5 when testing the bee-only data. There was no correlation between changes in species richness and species composition change in either dataset (electronic supplementary material, figure S2). No significant spatial autocorrelation was found between our sites in terms of changes in land cover, species richness change or species composition change.

### (c) Effects of land cover changes on species richness change

Details of the models showing the significant effects of different land cover types on species richness change are given in table 1, and the comparison of these models with the null model is given in electronic supplementary material, table S4. When testing for land cover effects on the full dataset (bees and wasps), the heathland change and woodland change at site and change in urban land at 1 km radius were found to have significant effects (table 1, models A1 and A2). The best model in terms of AICc showed that the most significant factor influencing species richness change was the change in edge habitat between urban land and grassland at the 1 km radius (table 1, model A3). Models A1, A2 and A3 explained 70.2%, 82.2% and 76.7%, respectively, of the heterogeneity in the data when compared with the null model.

**Table 1.** Results of the models showing the effect of changes in land cover on species richness.

response data	model no	significant land cover factors	estimate	*p*-value	ΔAICc	VAF^a^ (%)
models examining the effect of weighted land-use change on species richness						
complete dataset (bees and wasps)	A1	change in heathland at site	−0.0009	0.05	−0.20	70.16
		change in urban land at 1 km radius	0.0137	<0.01		
	A2	change in woodland at site	0.0011	<0.05	−8.26	82.21
		change in urban land at 1 km radius	0.0124	<0.01		
bees only	B1	change in urban land at 1 km radius	0.013	<0.01	1.61	54.73
models examining the effect of edge density change on species richness						
complete dataset (bees and wasps)	A3	urban land–grassland at 1 km radius	0.017	<0.001	6.24	76.64
bees only	B2	woodland–grassland at site	0.0054	<0.01	4.44	97.89
		woodland–other at site	0.0568	<0.05		
		heathland–grassland at 1 km radius	0.0143	<0.01		
		arable-other at 1 km radius	−0.0408	<0.05		

^a^VAF is the amount of heterogeneity accounted for by the model when compared with the null model.

The change in urban land at 1 km radius was found to have a significant positive effect on change in bee species richness (table 1, model B1), explaining 54.7% of the heterogeneity when compared with the null model. In comparison, model B2 explained 97.9% of heterogeneity in the data, and showed that the change in edge habitat between woodland and grassland, and woodland and other habitat at site, as well as the change in edge habitat between heathland and grassland at 1 km, had a positive impact on change in species richness, whereas the increase in edge habitat between arable land and other habitat at 1 km radius had a negative impact on change in bee species richness. There was no significant effect of change in habitat suitability at any scale, nor was there a significant effect of total change in land cover type or edge habitat changes at the 2, 5 and 10 km radii on species richness change.

### (d) Effects of land cover changes on species composition change

There was no significant effect of overall land-cover change on species composition change (table 2, models 1A and 1B). The edge habitat models indicated that the change in the grassland–arable edge habitat at the site level significantly influenced both bee and wasp species composition change and bee-only species composition change (table 2, models 2A and 2B). In addition, the change in edge habitat between heathland and woodland at the 1 km radius was found to significantly affect the changes in bee and wasp species composition (model 2A). As with species richness change models, land cover change at the 2, 5 and 10 km radii were not found to have any significant effect on species composition change, nor was there any significant impact of change in habitat suitability at any scale.

**Table 2.** Results of the models showing the effect of changes in land cover on species composition.

response data	model no	significant land cover factors	estimate	*p*-value	ΔAICc
models examining the effect of weighted land-use change on species composition					
complete dataset (bees and wasps)	1A	change in urban land at 1 km radius	−0.0118	0.07	0.46
bees only	1B	change in heathland at 1 km radius	0.0034	0.06	−0.20
		change in urban land at 1 km radius	−0.0117	0.08	
models examining the effect of edge density change on species composition					
complete dataset (bees and wasps)	2A	grassland–arable land at site	0.0197	<0.01	9.03
		heathland–woodland at 1 km radius	−0.0223	<0.05	
bees only	2B	grassland–arable land at site	0.0227	<0.05	3.96

## 4. Discussion

Over the past decade, the impact of landscape on insect pollinator communities, in terms of both scale and heterogeneity, has received much attention [12,13,22,42,43]. Most (if not all) of these studies have, however, relied on contemporary data using space-for-time substitutions in order to draw their conclusions. Our study is the first to use historical data to explore the impact that changing landscapes have had on pollinator richness and community composition. By analysing how anthropogenic activities have influenced the trends in pollinators over the past 80 years, our findings can aid informing current policy and practice with regard to future land management.

Our results show that 75% of our study sites saw a significant decline in species richness of both bees and wasps. However, there was no significant correlation observed between species richness change and species composition change. Changes in species composition could therefore be due to (i) loss of species, (ii) new arrivals/gains of species or (iii) a combination of species gains and losses.

We found that changes in both richness and composition of species were influenced by land cover changes within site as well as changes in the surrounding landscape. Sites surrounded primarily by arable expansion showed a greater decline in species richness than sites that did not, and this result concurs with previous studies showing that agricultural intensification with large monocultures of crops have led to significant declines in pollinator numbers [13,44,45]. While the increased use of herbicides and pesticides in modern agriculture has almost certainly had a role to play in driving pollinator declines [11,22,45], the lack of historical data on chemical input (or lack thereof) means this aspect could not be explored further.

Declines in pollinators in arable environments may have serious consequences in terms of loss of crop pollination services. However, studies have shown these losses can be ameliorated by the presence of heterogeneous landscapes, which include, for example, flower-rich meadows, hedgerows, woodland and other semi-natural habitat surrounding arable fields that provide foraging and nesting resources for pollinators [13,45–48]. While these studies support our result that heterogeneous landscapes are better for pollinators, it also has wider implications in terms of policy on how landscapes should be managed and the implementation of future agri-environment schemes.

In direct contrast to sites surrounded by arable intensification, sites surrounded by landscapes with urban expansion have proportionally lost fewer species. Previous studies have shown that urban areas can support diverse pollinator assemblages, but this capacity is strongly affected by local habitat quality [16,49]. In addition, a recent study has shown that urban environments support higher richness of bees in general and bumblebees in particular when compared with farmland and nature reserves [50]. This could be because urban areas (including recreation park spaces, gardens and churchyards) could provide diverse and extended forage, as well as provide nesting habitats, which might, in turn, promote pollinator richness and abundance. Some studies have suggested that pollinators can thrive in human-dominated landscapes [51], and although most of our sites showed declines in species richness, the loss of fewer species in sites surrounded by urban expansion shows that urban spaces could possibly provide a buffer against the changes within site, thereby curbing loss of species.

While our study sites were historically predominantly heathland, in agreement with previous studies [48,52], sites with increased woodland area showed a positive correlation with change in species richness. Historical research has emphasized the influence of habitat edges on increased species richness [53–55], and the transitional habitat between heathland and woodland would in effect increase such edge habitat, potentially providing more diverse nesting as well as forage resources. Our results therefore support the theory that complex heterogeneous landscapes are conducive to greater pollinator diversity.

Our study confirms previous research that both scale and heterogeneity of landscape need to be considered when planning for land management [56]. It is not just changes within a site that need to be considered, but also changes in the wider landscape context at spatial scales relevant to pollinators. For example, studies that have looked at the impact of agri-environment schemes in Britain aimed at improving pollinators and ecosystem services have suggested how well-designed, cooperative landscape-level management plans might be more beneficial and effective than farm-level schemes [57]. Similarly, the importance of habitat diversity in the surrounding landscape and inclusion of non-agricultural habitats within land management plans have been shown to boost pollinator numbers, thereby improving ecosystem services and yield of economically important crops such as oil seed rape, field beans, strawberries, buckwheat and cherry [42,46,52].

Our study highlights the value of historical records as a research resource that can be used to inform land management to conserve biodiversity. While more detailed research is required on specific land management practices that can support and enhance pollinator diversity (and thereby impact crop yields), large-scale landscape-level manipulations are not always feasible; our study therefore serves as a vital source of information on the impact of landscape-level transformation of habitat types on insect pollinators. The timing of our study means it has the potential to have national-level influence, especially in the light of changing agri-environment policy and the New Environmental Land Management Scheme, by providing information that could be used for future policy related to land management for ecosystem services and food security.

## Supplementary Material

ESM for ‘The impact of over 80 years of land cover changes on bee and wasp pollinator communities in England’

## Data accessibility

Data on insect pollinators are available from the database of the Bees, Wasps and Ants Recording Society (BWARS), accessible via the National Biodiversity Network (NBN) website at http://www.nbn.org.uk/. Land-cover data are available via the CEH Information Gateway (https://gateway.ceh.ac.uk/home).

## References

[1] Biesmeijer JC 2006 Parallel declines in pollinators and insect-pollinated plants in Britain and the Netherlands. Science 313, 351–354. (10.1126/science.1127863) 16857940

[2] Butchart SHM 2010 Global biodiversity: indicators of recent declines. Science 328, 1164–1168. (10.1126/science.1187512) 20430971

[3] Lebuhn G 2013 Detecting insect pollinator declines on regional and global scales. Conserv. Biol. 27, 113–120. (10.1111/j.1523-1739.2012.01962.x) 23240651

[4] Bommarco R Lundin O Smith HG Rundlöf M 2011 Drastic historic shifts in bumble-bee community composition in Sweden. Proc. R. Soc. B 279, 309–315. (10.1098/rspb.2011.0647) PMC3223670 21676979

[5] Carvalheiro LG 2013 Species richness declines and biotic homogenisation have slowed down for NW European pollinators and plants. Ecol. Lett. 16, 870–878. (10.1111/ele.12121) 23692632 PMC3738924

[6] Hegland SJ Nielsen A Lázaro A Bjerknes N Totland Ø 2009 How does climate warming affect plant–pollinator interactions? Ecol. Lett. 12, 184–195. (10.1111/j.1461-0248.2008.01269.x) 19049509

[7] Memmott J Craze PG Waser NM Price MV 2007 Global warming and the disruption of plant–pollinator interactions. Ecol. Lett. 10, 710–717. (10.1111/j.1461-0248.2007.01061.x) 17594426

[8] Cameron SA Lozier JD Strange JP Koch JB Cordes N Solter LF Griswold TL 2011 Patterns of widespread decline in North American bumble bees. Proc. Natl Acad. Sci. USA 108, 662–667. (10.1073/pnas.1014743108) 21199943 PMC3021065

[9] Abe T Makino S Okochi I 2008 Why have endemic pollinators declined on the Ogasawara Islands? Biodivers. Conserv. 17, 1465–1473. (10.1007/s10531-008-9355-y)

[10] Moron D Lenda M Skorka P Szentgyorgyi H Settele J Woyciechowski M 2009 Wild pollinator communities are negatively affected by invasion of alien goldenrods in grassland landscapes. Biol. Conserv. 142, 1322–1332. (10.1016/j.biocon.2008.12.036)

[11] Kremen C Williams NM Thorp RW 2002 Crop pollination from native bees at risk from agricultural intensification. Proc. Natl Acad. Sci. USA 99, 16 812–16 816. (10.1073/pnas.262413599) PMC139226 12486221

[12] Tscharntke T Klein AM Kruess A Steffan-Dewenter I Thies C 2005 Landscape perspectives on agricultural intensification and biodiversity: ecosystem service management. Ecol. Lett. 8, 857–874. (10.1111/j.1461-0248.2005.00782.x)

[13] Kennedy CM 2013 A global quantitative synthesis of local and landscape effects on wild bee pollinators in agroecosystems. Ecol. Lett. 16, 584–599. (10.1111/ele.12082) 23489285

[14] Garibaldi LA 2011 Stability of pollination services decreases with isolation from natural areas despite honey bee visits. Ecol. Lett. 14, 1062–1072. (10.1111/j.1461-0248.2011.01669.x). 21806746

[15] Winfree R Bartomeus I Cariveau DP 2011 Native pollinators in anthropogenic habitats. Ann. Rev. Ecol. Evol. Syst. 42, 1–22.

[16] Bates AJ Sadler JP Fairbrass AJ Falk SJ Hale JD Matthews TJ 2011 Changing bee and hoverfly pollinator assemblages along an urban–rural gradient. PLoS ONE 6, e23459 (10.1371/journal.pone.0023459) 21858128 PMC3155562

[17] Cariveau DP Williams NM Benjamin FE Winfree R 2013 Response diversity to land use occurs but does not consistently stabilise ecosystem services provided by native pollinators. Ecol. Lett. 16, 903–911. (10.1111/ele.12126) 23692675

[18] Goulson D Lepais O O'Connor S Osborne JL Sanderson RA Cussans J Goffe L Darvill B 2010 Effects of land use at a landscape scale on bumblebee nest density and survival. J. Appl. Ecol. 47, 1207–1215. (10.1111/j.1365-2664.2010.01872.x)

[19] Jha S Kremen C 2013 Urban land use limits regional bumble bee gene flow. Mol. Ecol. 22, 2483–2495. (10.1111/mec.12275) 23495763

[20] Matteson KC Grace JB Minor ES 2013 Direct and indirect effects of land use on floral resources and flower-visiting insects across an urban landscape. Oikos 122, 682–694. (10.1111/j.1600-0706.2012.20229.x)

[21] Steffan-Dewenter I Westphal C 2008 The interplay of pollinator diversity, pollination services and landscape change. J. Appl. Ecol. 45, 737–741. (10.1111/j.1365-2664.2008.01483.x)

[22] Vanbergen AJ, Insect Pollinators Initiative. 2013 Threats to an ecosystem service: pressures on pollinators. Front. Ecol. Environ. 11, 251–259. (10.1890/120126)

[23] Stamp LD 1948 The land of Britain: its use and misuse. London, UK: Longmans, Green and Co.

[24] Chao A Chazdon RL Colwell RK Shen TJ 2005 A new statistical approach for assessing similarity of species composition with incidence and abundance data. Ecol. Lett. 8, 148–159. (10.1111/j.1461-0248.2004.00707.x)

[25] Colwell RK Chao A Gotelli NJ Lin S-Y Mao CX Chazdon RL Longino JT 2012 Models and estimators linking individual-based and sample-based rarefaction, extrapolation and comparison of assemblages. J. Plant Ecol. 5, 3–21. (10.1093/jpe/rtr044)

[26] Klein AM Vaissiere BE Cane JH Steffan-Dewenter I Cunningham SA Kremen C Tscharntke T 2007 Importance of pollinators in changing landscapes for world crops. Proc. R. Soc. B 274, 303–313. (10.1098/rspb.2006.3721) PMC1702377 17164193

[27] Westphal C Steffan-Dewenter I Tscharntke T 2006 Bumblebees experience landscapes at different spatial scales: possible implications for coexistence. Oecologia 149, 289–300. (10.1007/s00442-006-0448-6) 16758219

[28] Fagan WF Cantrell RS Cosner C 1999 How habitat edges change species interactions. Am. Nat. 153, 165–182. (10.1086/303162) 29578760

[29] Morton RD Rowland C Wood C Meek L Marston C Smith G Wadsworth R Simpson IC 2011 Final report for LCM2007–the new UK land cover map. CS technical report no 11/07 Bailrigg, UK: Centre for Ecology and Hydrology.

[30] Greenleaf SS Williams NM Winfree R Kremen C 2007 Bee foraging ranges and their relationship to body size. Oecologia 153, 589–596. (10.1007/s00442-007-0752-9) 17483965

[31] Steffan-Dewenter I Tscharntke T 1999 Effects of habitat isolation on pollinator communities and seed set. Oecologia 121, 432–440. (10.1007/s004420050949) 28308334

[32] Walther-Hellwig K Frankl R 2000 Foraging habitats and foraging distances of bumblebees, *Bombus* spp. (Hym., Apidae), in an agricultural landscape. J. Appl. Entomol. 124, 299–306. (10.1046/j.1439-0418.2000.00484.x)

[33] ESRI. 2011 ArcGIS desktop: release 10. Redlands, CA: Environmental Systems Research Institute.

[34] Vogiatzakis IN Stirpe MT Rickebusch S Metzger MJ Xu G Rounsevell MDA Bommarco R Potts SG 2015 Rapid assessment of historic, current and future habitat quality for biodiversity around UK Natura 2000 sites. Environ. Conserv. 42, 31–40. (10.1017/S0376892914000137)

[35] McGarigal K Cushman SA Ene E 2012 Spatial pattern analysis program for categorical and continuous maps. Computer software program produced by the authors at the University of Massachusetts, Amherst. FRAGSTATS v4. See http://wwwumassedu/landeco/research/fragstats/fragstatshtml.

[36] Lennon JJ Koleff P Greenwood JJD Gaston KJ 2001 The geographical structure of British bird distributions: diversity, spatial turnover and scale. Ecology 70, 966–979.

[37] Paradis E Claude J Strimmer K 2004 APE: analyses of phylogenetics and evolution in R language. Bioinformatics 20, 289–290. (10.1093/bioinformatics/btg412) 14734327

[38] Viechtbauer W 2010 Conducting meta-analyses in R with the metafor package. J. Stat. Softw. 36, 1–48.

[39] Hartung J Knapp G Sinha BK 2008 Statistical meta-analysis with applications. Hoboken, NJ: John Wiley & Sons, Inc.

[40] Venables WN Ripley BD 2002 Modern applied statistics with S. Fourth edition New York, NY: Springer.

[41] R Development Core Team. 2013 R: a language and environment for statistical computing. Vienna, Austria: R Foundation for Statistical Computing See http://wwwR-projectorg.

[42] Bartomeus I 2014 Contribution of insect pollinators to crop yield and quality varies with agricultural intensification. PeerJ 2, e328 (10.7717/peerj.328) 24749007 PMC3976118

[43] Jha S Stefanovich L Kremen C 2013 Bumble bee pollen use and preference across spatial scales in human-altered landscapes. Ecol. Entomol. 38, 570–579. (10.1111/een.12056)

[44] Ferreira PA Boscolo D Viana BF 2013 What do we know about the effects of landscape changes on plant-pollinator interaction networks? Ecol. Indic. 31, 35–40. (10.1016/j.ecolind.2012.07.025)

[45] Nicholls CI Altieri MA 2013 Plant biodiversity enhances bees and other insect pollinators in agroecosystems: a review. Agron. Sustain. Dev. 33, 257–274. (10.1007/s13593-012-0092-y)

[46] Osgathorpe LM Park K Goulson D 2012 The use of off-farm habitats by foraging bumblebees in agricultural landscapes: implications for conservation management. Apidologie 43, 113–127. (10.1007/s13592-011-0083-z)

[47] Morandin LA Kremen C 2013 Hedgerow restoration promotes pollinator populations and exports native bees to adjacent fields. Ecol. Appl. 23, 829–839. (10.1890/12-1051.1) 23865233

[48] Patricio-Roberto GB Campos MJO 2014 Aspects of landscape and pollinators: what is important to bee conservation? Diversity-Basel 6, 158–175. (10.3390/d6010158)

[49] Hinners SJ Kearns CA Wessman CA 2012 Roles of scale, matrix, and native habitat in supporting a diverse suburban pollinator assemblage. Ecol. Appl. 22, 1923–1935. (10.1890/11-1590.1) 23210309

[50] Baldock K 2015 Where is the UK's pollinator biodiversity? Comparing flower-visitor communities between cities, farmland and nature reserves using visitation networks. Proc. R. Soc. B 282, 20142849 (10.1098/rspb.2014.2849)

[51] Bartomeus I Winfree R 2013 Pollinator declines: reconciling scales and implications for ecosystem services. F1000Research 2, 146 (10.12688/f1000research.2-146.v1) 24555067 PMC3892917

[52] Schueepp C Herzog F Entling MH 2014 Disentangling multiple drivers of pollination in a landscape-scale experiment. Proc. R. Soc. B 281, 20132667 (10.1098/rspb.2013.2667) PMC3843845 24225465

[53] Odum EP 1971 Fundamentals of ecology. Philadelphia, PA: WB Sanders Co.

[54] Leopold A 1933 Game management. New York, NY: Charles Scribner's Sons.

[55] Kunin WE 1998 Biodiversity at the edge: a test of the importance of spatial ‘mass effects’ in the Rothamsted Park grass experiments. Proc. Natl Acad. Sci. USA 95, 207–212. (10.1073/pnas.95.1.207) 9419354 PMC18177

[56] Westrich P 1996 Habitat requirements of central European bees and the problems of partial habitat. In The conservation of bees (eds Matheson A Buchman SL O'Toole C Westrich P Williams IH), pp. 1–16. London, UK: Academic Press.

[57] McKenzie AJ Emery SB Franks JR Whittingham MJ 2013 Landscape-scale conservation: collaborative agri-environment schemes could benefit both biodiversity and ecosystem services, but will farmers be willing to participate? J. Appl. Ecol. 50, 1274–1280. (10.1111/1365-2664.12122)

