# Supplementary material for: The impact of over 80 years of land cover changes on bee and wasp pollinator communities in England
Source: Proc Biol Sci. 2015 May 7;282(1806):20150294. doi: 10.1098/rspb.2015.0294 (PMC4426632; doi:10.1098/rspb.2015.0294)
Supplement: ESM for ‘The impact of over 80 years of land cover changes on bee and wasp pollinator communities in England’ [file rspb20150294supp1.docx]

S**upplementary material**

**The impact of over 80 years of land cover changes on bee and wasp pollinator communities in England**

**Materials & methods**

*Study sites and field work:*

The 20 sites identified as having historic data were visited in 2011 and 2012 and resampled three times between April and August using a combination of pan traps (five sets of one blue, one white and one yellow put out from morning until evening, including the core activity hours of 10am-4pm) and timed transects (4 person hours per site, per round). While the field methods used historically are unknown, but probably comprise mainly of unrestricted collecting with nets (Stuart Roberts, pers. comm.), we have applied two of the most effective and widely used sampling methods (Westphal. et al. 2008) systematically to all our re-observation sites.

**Supplementary Table 1: Location and details of the 20 study sites**

| **Site Name** | **Site Code** | **Historic County** | **Latitude** | **Longitude** | **Predominant historic land cover** | **Current Status** |
| --- | --- | --- | --- | --- | --- | --- |
| Aspley Heath | B-AH | Bedfordshire | 52° 0'12.30"N | 0°38'46.63"W | Woodland (93%) | SSSI |
| Deadmansea Wood | B-DW | Bedfordshire | 51°50'22.76"N | 0°29'53.39"W | Woodland (67%) | Private woodland |
| Flitwick Moor | B-FM | Bedfordshire | 52° 0'16.71"N | 0°28'21.73"W | Heathland (70%) | SSSI |
| King's Wood | B-HR | Bedfordshire | 51°57'16.35"N | 0°39'18.90"W | Woodland (94%) | SSSI, NNR |
| Totternhoe Knoll | B-TT | Bedfordshire | 51°53'19.50"N | 0°34'43.77"W | Heathland (44%) | SSSI, NNR |
| Whipsnade Heath | B-WH | Bedfordshire | 51°51'5.85"N | 0°31'30.25"W | Heathland (85%) | - |
| Wicken Fen | C-WF | Cambridgeshire | 52°18'38.25"N | 0°17'28.22"E | Heathland (81%) | SSSI, NNR |
| Gore Heath | D-GH | Dorset | 50°43'19.08"N | 2° 6'33.90"W | Heathland (68%) | FC |
| Godlingston Heath | D-GN | Dorset | 50°38'33.17"N | 1°58'12.28"W | Heathland (98%) | SSSI, NNR |
| Holt Heath | D-HH | Dorset | 50°50'16.95"N | 1°54'49.78"W | Heathland (76%) | SSSI, NNR |
| Lyme Regis | D-LR | Dorset | 50°43'58.69"N | 2°55'22.68"W | Heathland (53%) | SSSI |
| Morden Heath | D-MH | Dorset | 50°43'50.55"N | 2° 7'34.60"W | Heathland (56%) | SSSI, NNR |
| Rempstone Heath | D-RH | Dorset | 50°38'55.75"N | 1°59'28.74"W | Heathland (92%) | FC |
| Studland Heath | D-SH | Dorset | 50°39'39.28"N | 1°57'5.66"W | Heathland (94%) | SSSI, NNR |
| Allerthorpe Common | L-AC | Yorkshire | 53°55'10.77"N | 0°50'50.90"W | Heathland (97%) | SSSI, NR |
| Barmby Moor | L-BM | Yorkshire | 53°56'16.63"N | 0°51'24.41"W | Heathland (44%) | NR |
| Harden Moor | L-HR | Yorkshire | 53°50'42.21"N | 1°53'30.57"W | Heathland (86%) | NR |
| Roundhay Park | L-RH | Yorkshire | 53°50'14.37"N | 1°29'58.37"W | Grassland (84%) | City Park |
| Shipley Glen | L-SG | Yorkshire | 53°50'50.98"N | 1°48'10.59"W | Heathland (75%) | - |
| Spurn Point | L-SP | Yorkshire | 53°34'26.46"N | 0° 6'34.15"E | Heathland (86%) | SSSI |

FC – Forestry Commission Land; NNR – National Nature Reserve; NR – Nature Reserve; SAC – Special Area of Conservation; SSSI – Site of Special Scientific Interest

*Reclassification of land cover maps*

**Supplementary Table 2: Reclassification of LCM 2007 broad habitat categories and Dudley Stamp land cover categories**

| **LCM 2007 Original Broad Habitat Categories & Values** | | **Corresponding Dudley Stamp Categories and Values** | **Reclassified Land Cover Category** |
| --- | --- | --- | --- |
| 1 | Broad leaved, mixed & Yew woodland | Forest & Woodland | Woodland |
| 2 | Coniferous woodland | Forest & Woodland | Woodland |
| 3 | Arable & Horticulture | Arable | Arable Land |
| 4 | Improved Grassland | Meadow & Grassland | Grassland |
| 5 | Rough Grassland | Meadow & Grassland | Grassland |
| 6 | Neutral grassland | Meadow & Grassland | Grassland |
| 7 | Calcareous grassland | Meadow & Grassland | Grassland |
| 8 | Acid grassland | Meadow & Grassland | Grassland |
| 9 | Fen, Marsh & Swamp | Heath & Moorland | Heathland |
| 10 | Heather | Heath & Moorland | Heathland |
| 11 | Heather grassland | Heath & Moorland | Heathland |
| 12 | Bog | Heath & Moorland | Heathland |
| 13 | Montane habitats | Heath & Moorland | Heathland |
| 14 | Inland Rock | Other | Other |
| 15 | Saltwater | Water | Other |
| 16 | Freshwater | Water | Other |
| 17 | Supra-littoral rock | Other | Other |
| 18 | Supra-littoral sediment | Heath & Moorland | Heathland |
| 19 | Littoral rock | Other | Other |
| 20 | Littoral sediment | Heath & Moorland | Heathland |
| 21 | Saltmarsh | Other | Other |
| 22 | Suburban | Suburban | Urban |
| 23 | Urban | Urban | Urban |
| 24 | NA | Orchards & Gardens | Urban |

*Note:* Urban, suburban and orchard categories were grouped together into one ‘urban’ category due to the variations in definitions between the two maps. As the extent of classified coastal waters varied between the two maps, water and other non-classified categories were also grouped into one ‘other’ category for further analysis.

*Pollinator suitability score:*

We used the pollinator suitability score as determined for each land cover type by European experts on the COCONUT project (Vogiatzakis et al. 2015). These scores were also utilised to calculate a change in habitat suitability for each site at each spatial scale as follows to be included in further analyses.

$Change in habitat suitability=\ln\left[ \frac{{Current Habitat Suitability}}{Historic Habitat Suitability} \right]$ where

*Historic Habitat suitability= ∑ (% of each historic land cover category x pollinator suitability score for that category)*

*Current Habitat Suitability = ∑ (% of each current land cover category x pollinator suitability score for that category)*

*Analyses of land cover change on change in species richness*

Changes in land cover types that were correlated with each other were excluded from being in the same model (for example, heathland and woodland which are negatively correlated with each other, were not tested within the same model but each were tested in models combined with other non-correlated land cover variables). Changes in edge habitat were tested in separate models as edge density change is correlated with overall change in each habitat type. Land cover changes at different spatial scales outside of the site were tested in separate models (for example Model 1= land cover change at site + change at 1km; Model 2 = land cover change at site + change at 2km; Model 3 = Edge habitat at site +edge habitat at 1km radius; Model 4 = Edge habitat at site +edge habitat at 2km radius etc.) and all possible interactions between land cover variables were also tested. Each model was simplified using a step AIC method until only the minimum adequate model remained. The models showing significant land cover change variables were then compared to the null model to determine what percentage of the existing heterogeneity could be explained by the inclusion of the explanatory variables.

**Results:**

**
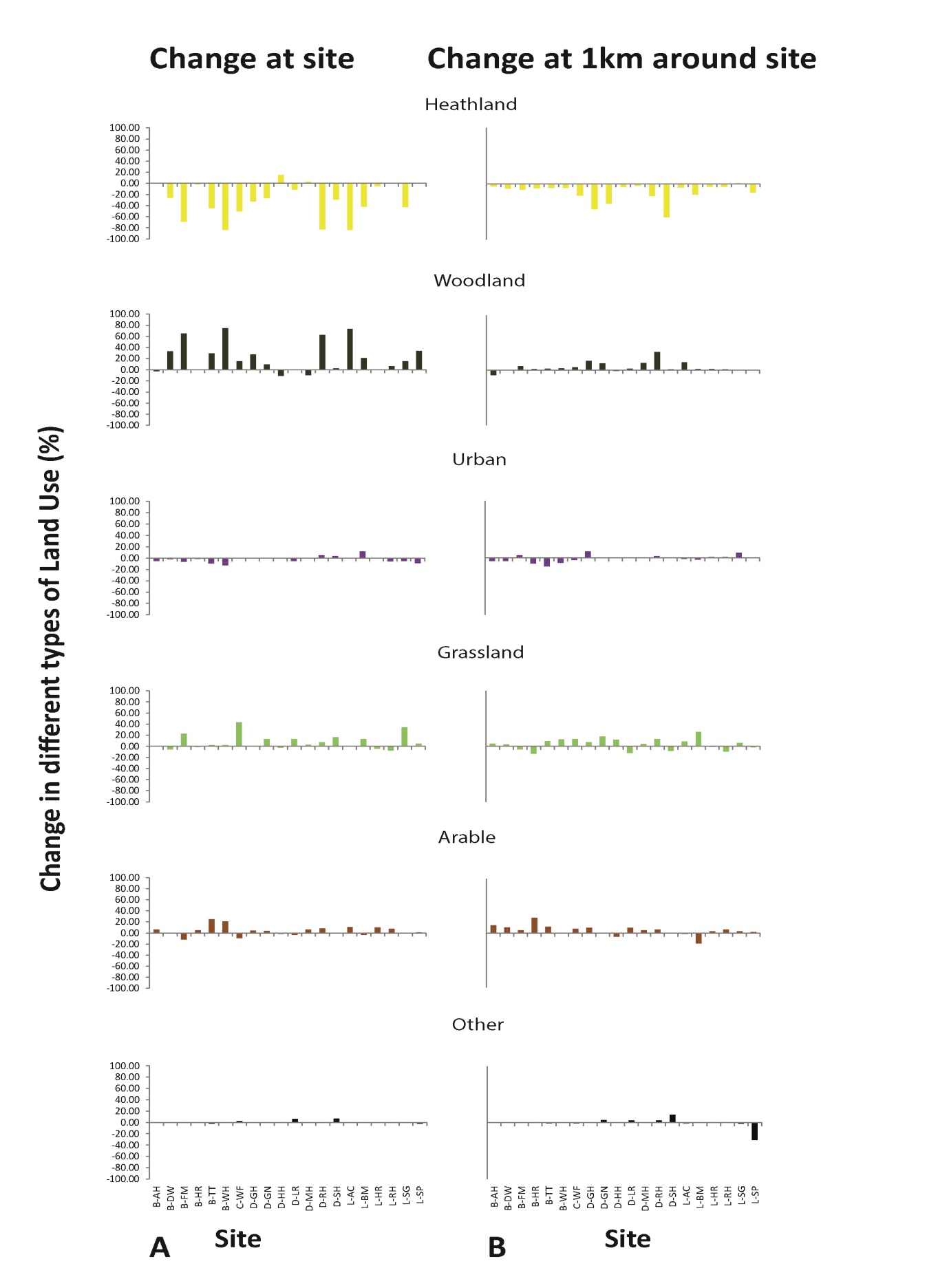
**

**Supplementary Figure 1: Change in different land cover types (%) at site (column A) and around the site at 1km radius (column B)**

**Supplementary Table 3: Percentage change in land cover classes at 2km, 5km and 10km radii outside of the sites**

| **Percentage change in land cover classes at 2km buffer around each site** | | | | | | | |
| --- | --- | --- | --- | --- | --- | --- | --- |
| **County** | **Site** | **Heathland** | **Woodland** | **Arable** | **Grassland** | **Urban** | **Other** |
| Bedfordshire | Aspley Heath | -4.61 | -0.96 | 17.6 | -5.39 | -6.15 | -0.48 |
| Bedfordshire | Deadmansea Wood | -3.33 | 3.35 | 32.47 | -21.98 | -9.8 | -0.71 |
| Bedfordshire | Flitwick Moor | -4.29 | 2.64 | 10.14 | -11.75 | 4.29 | -1.04 |
| Bedfordshire | King's wood | -5.56 | 3.70 | 27.98 | -17.19 | -9.09 | 0.16 |
| Bedfordshire | Totternhoe | -3.02 | 1.33 | 5.39 | 6.74 | -11.98 | 1.54 |
| Bedfordshire | Whipsnade Heath | -5.38 | 3.95 | 14.53 | 0.69 | -13.5 | -0.28 |
| Cambridgeshire | Wicken Fen | -7.3 | 1.84 | 17.56 | -7.39 | -3.41 | -1.29 |
| Dorset | Gore Heath | -36.88 | 14.95 | 0.34 | 9.42 | 12 | 0.17 |
| Dorset | Godlingston Heath | -27.6 | 6.85 | 0.07 | 16.06 | -1.87 | 6.48 |
| Dorset | Holt Heath | -22.57 | 3.51 | -1.03 | 11.65 | 8.32 | 0.12 |
| Dorset | Lyme Regis | -4.87 | 4.87 | 12.95 | -13.74 | -1.43 | 2.23 |
| Dorset | Morden Heath | -28.26 | 19.29 | 5.02 | 4.67 | -0.57 | -0.15 |
| Dorset | Rempstone Heath | -39.53 | 14.86 | 1.70 | 12.82 | 1.76 | 8.39 |
| Dorset | Studland Heath | -3.96 | 3.92 | 0.86 | -13.75 | -1.44 | 14.37 |
| Yorkshire | Allerthorpe Common | -2.23 | 0.56 | 5.08 | -3.20 | 0.26 | -7.31 |
| Yorkshire | Barmby Moor | -12.21 | 8.17 | -2.75 | 9.87 | -2.61 | -0.47 |
| Yorkshire | Harden Moor | -6.09 | 6.55 | 2.65 | -9.43 | 7.62 | -1.31 |
| Yorkshire | Round Hay Park | 0.72 | -0.74 | -0.85 | -13.54 | 15.01 | -0.60 |
| Yorkshire | Shipley Glen | -9.87 | 1.04 | 1.01 | -2.45 | 11.91 | -1.64 |
| Yorkshire | Spurn point | 20.83 | -0.01 | 1.45 | -1.58 | -0.5 | -20.19 |
| **Percentage change in land cover classes at 5km buffer around each site** | | | | | | | |
| **County** | **Site** | **Heathland** | **Woodland** | **Arable** | **Grassland** | **Urban** | **Other** |
| Bedfordshire | Aspley Heath | -0.47 | 1.47 | 23.74 | -34.88 | 9.65 | 0.49 |
| Bedfordshire | Deadmansea Wood | -1.00 | 1.88 | 16.02 | -17.71 | 0.72 | 0.09 |
| Bedfordshire | Flitwick Moor | -1.37 | 1.92 | 27.56 | -25.21 | -2.13 | -0.77 |
| Bedfordshire | King's wood | -2.49 | 1.42 | 30.09 | -24.45 | -3.41 | -1.16 |
| Bedfordshire | Totternhoe | -1.11 | 1.54 | 26.62 | -20.59 | -6.88 | 0.44 |
| Bedfordshire | Whipsnade Heath | -3.20 | 3.27 | 9.96 | -7.52 | -2.78 | 0.29 |
| Cambridgeshire | Wicken Fen | -2.75 | 0.78 | 15.36 | -10.38 | -2.09 | -0.91 |
| Dorset | Gore Heath | -21.58 | 3.35 | 5.77 | 0.13 | 3.89 | 8.43 |
| Dorset | Godlingston Heath | -9.49 | 3.90 | -1.25 | 1.02 | 1.80 | 4.02 |
| Dorset | Holt Heath | -23.47 | 3.73 | 8.84 | 2.31 | 8.88 | -0.30 |
| Dorset | Lyme Regis | -9.29 | 6.23 | 14.84 | -8.59 | -3.55 | 0.37 |
| Dorset | Morden Heath | -24.56 | 11.77 | 10.27 | -2.21 | 1.00 | 3.74 |
| Dorset | Rempstone Heath | -24.08 | 6.65 | 2.50 | 4.71 | 0.51 | 9.71 |
| Dorset | Studland Heath | -3.15 | -8.18 | 0.54 | -7.57 | 14.39 | 3.97 |
| Yorkshire | Allerthorpe Common | -0.72 | -0.32 | 17.16 | -13.41 | -0.64 | -2.06 |
| Yorkshire | Barmby Moor | -3.03 | 2.18 | 13.75 | -9.81 | -1.32 | -1.78 |
| Yorkshire | Harden Moor | -3.53 | 3.50 | 2.07 | -6.98 | 6.62 | -1.69 |
| Yorkshire | Round Hay Park | -0.14 | 0.93 | -1.94 | -10.38 | 12.13 | -0.61 |
| Yorkshire | Shipley Glen | -4.77 | 2.32 | 0.09 | -7.19 | 10.95 | -1.38 |
| Yorkshire | Spurn point | 10.45 | -0.07 | 1.84 | -2.29 | -0.11 | -9.83 |
| **Percentage change in land cover classes at 10km buffer around each site** | | | | | | | |
| **County** | **Site** | **Heathland** | **Woodland** | **Arable** | **Grassland** | **Urban** | **Other** |
| Bedfordshire | Aspley Heath | -0.30 | 0.87 | 17.26 | -39.51 | 21.09 | 0.58 |
| Bedfordshire | Deadmansea Wood | -1.54 | 0.69 | -3.20 | -10.39 | 15.31 | -0.86 |
| Bedfordshire | Flitwick Moor | -1.30 | 2.36 | 29.06 | -28.91 | -2.17 | 0.26 |
| Bedfordshire | King's wood | -1.21 | 0.29 | 25.49 | -33.06 | 8.89 | -0.40 |
| Bedfordshire | Totternhoe | -2.18 | 2.62 | 40.01 | -36.18 | -3.52 | -0.75 |
| Bedfordshire | Whipsnade Heath | -4.22 | 3.84 | 12.49 | -11.14 | -0.78 | -0.19 |
| Cambridgeshire | Wicken Fen | -1.41 | 0.19 | 12.18 | -7.72 | -2.59 | -0.66 |
| Dorset | Gore Heath | -22.09 | 7.88 | 4.11 | 2.01 | -2.92 | 11.01 |
| Dorset | Godlingston Heath | -0.26 | 0.20 | -0.01 | -1.05 | -1.51 | 2.63 |
| Dorset | Holt Heath | -14.97 | 1.55 | 10.59 | -3.48 | 7.18 | -0.87 |
| Dorset | Lyme Regis | -4.76 | 2.96 | 18.57 | -13.04 | -4.37 | 0.64 |
| Dorset | Morden Heath | -20.87 | 8.87 | 13.02 | -4.77 | 2.21 | 1.53 |
| Dorset | Rempstone Heath | -9.23 | -0.21 | 4.26 | -0.83 | 2.61 | 3.40 |
| Dorset | Studland Heath | -0.46 | -0.06 | 0.17 | -5.75 | 1.47 | 4.63 |
| Yorkshire | Allerthorpe Common | -0.46 | -0.09 | 30.19 | -26.35 | -2.38 | -0.91 |
| Yorkshire | Barmby Moor | -1.53 | 1.06 | 21.09 | -18.12 | -1.57 | -1.73 |
| Yorkshire | Harden Moor | -4.61 | 2.22 | 0.59 | -1.18 | 4.50 | -1.52 |
| Yorkshire | Round Hay Park | -0.40 | 1.87 | 5.78 | -13.99 | 7.87 | -1.14 |
| Yorkshire | Shipley Glen | -0.64 | 2.28 | -0.80 | -11.02 | 11.41 | -1.25 |
| Yorkshire | Spurn point | 12.32 | 0.04 | 5.53 | -5.77 | 0.68 | -12.80 |

**Supplementary Table 3: Results of comparison between the minimum adequate model and null model testing for the impacts of change in land cover on species richness change (SRC)**

**Bee & Wasp data**

Moderators AIC AICc logLik pval QE tau^2 VAF

Null Model None -8.46 -7.37 6.23 38.53 0.0135

Model A1 Site heathland, urban 1km -11.62 -7.17 9.81 0.02 21.09 0.004 70.16%

Model A2 Site woodland, urban 1km -11.00 0.89 11.50 0.03 16.93 0.002 82.21%

Model A3 Urban-grassland 1km edge -16.01 -13.61 11.01 0.002 18.56 0.003 76.64%

**Bees only data**

Moderators AIC AICc logLik pval QE tau^2 VAF

Null Model None -0.68 0.65 2.34 54.34 0.028

Model B1 Urban 1km -3.96 -0.96 4.98 0.02 26.13 0.013 54.73%

Model B2 Wood-grass site edge -20.59 -3.79 16.29 <0.001 67.77 0.001 97.89%

Wood-other site edge

Heath-grass 1km edge

Arable-other 1km edge

Pearson's correlation = -0.12

p = 0.6

**Supplementary Figure 2: Scatter plots showing correlation between change in species richness and change in species composition for the different datasets tested**

**References:**

Carvalheiro L.G., Kunin W.E., Keil P., Aguirre-Gutierrez J., Ellis W.N., Fox R., Groom Q., Hennekens S., Van Landuyt W., Maes D., et al. 2013 Species richness declines and biotic homogenisation have slowed down for NW-European pollinators and plants. *Ecol Lett* **16**(7), 870-878. (doi:10.1111/ele.12121).

Vogiatzakis I.N., Stirpe M.T., Rickebusch S., Metzger M.J., Xu G., Rounsevell M.D.A., Bommarco R., Potts S.G. 2015 Rapid assessment of historic, current and future habitat quality for biodiversity around UK Natura 2000 sites. Environ Conserv **42**(01), 31-40. (doi:doi:10.1017/S0376892914000137).

Westphal C., Steffan-Dewenter I., Tscharntke T. 2006 Bumblebees experience landscapes at different spatial scales: possible implications for coexistence. *Oecologia* **149**(2), 289-300. (doi:10.1007/s00442-006-0448-6).
